# The Influence of Annealing Temperature on the Morphology of Structures and the Mechanical Properties of Prequenching—Quenching and Partitioning Steel

**DOI:** 10.3390/ma15124156

**Published:** 2022-06-11

**Authors:** Deming Xu, Yuanyao Cheng, Gengwei Yang, Gang Zhao, Siqian Bao

**Affiliations:** 1 The State Key Laboratory of Refractories and Metallurgy, Wuhan University of Science and Technology, Wuhan 430081, China; xudeming@wust.edu.cn (D.X.); chengyuanyao2014@gmail.com (Y.C.); zhaogang@wust.edu.cn (G.Z.); baosiqian@wust.edu.cn (S.B.); 2 Key Laboratory for Ferrous Metallurgy and Resources Utilization of Ministry of Education, Wuhan University of Science and Technology, Wuhan 430081, China

**Keywords:** prequenching, quenching and partitioning treatment, annealing temperature, morphology of structures, total elongation

## Abstract

In this study, we comparatively study the microstructures and mechanical properties of prequenching—quenching and partitioning (QQ&P) and traditional Q&P samples at different annealing temperatures (intercritical annealing temperatures). When the annealing temperature is 780 °C, the ferrite and retained austenite in QQ&P samples with lath and blocky morphologies. The lath retained austenite is mainly distributed along the lath ferrite. As the annealing temperature increases, the lath ferrite recrystallizes and gradually grows into the blocky (equiaxed) shape, leading to a decrease in the lath retained austenite content. When the annealing temperature increases to 870 °C, the ferrite content decreases significantly, and the retained austenite is mainly blocky and thin film, distributed at the boundaries of prior austenite grains and between martensite laths, respectively. Different from QQ&P samples, the ferrite and retained austenite in Q&P samples are mainly blocky when the annealing temperature is 780 °C or 810 °C. When the annealing temperature is increased to 870 °C, the microstructures of the Q&P sample are basically the same as that of the QQ&P sample. The 780 °C-QQ&P sample and the 810 °C-QQ&P sample have higher total elongation and product of strength and elongations (PSEs) than their counterpart Q&P samples due to the fact that lath ferrite and retained austenite are conducive to carbon diffusion and carbon homogenization in austenite grains, thereby improving the thermal stability and volume fraction of the retained austenite. In addition, the lath structures can release local stress concentration and delay the formation of voids and microcracks. The difference of mechanical properties between QQ&P samples and Q&P samples decreases with the increase in the annealing temperature. The results show that the low annealing temperature combined with prequenching—Q&P heat treatments can significantly improve the elongation and PSE of Q&P steel.

## 1. Introduction

The demand for passenger safety and fuel efficiency in the global automobile industry has spurred the development of new and advanced high−strength steels (AHSS). The quenching and partitioning (Q&P) processing, initially proposed by Speer et al. [1,2], is a promising and innovative heat treatment to prepare third−generation AHSS. This treatment starts with a partial or full austenization followed by an interrupted quench at a temperature between the martensite start temperature (M_s_) and the martensite finish temperature (M_f_), and then a holding period at the same temperature or a higher one to migrate carbon from the supersaturated martensite into untransformed austenite. Austenite with a high carbon content can be retained at room temperature. The effects of such a treatment on mechanical properties depend strongly on the transformation−induced plasticity (TRIP) effect of the retained austenite.

The TRIP effect is generally believed to be controlled by the volume fraction and the stability of the retained austenite [3,4,5]. Improving the volume fraction of the retained austenite and maintaining appropriate mechanical stability through reasonable heat treatment can help enhance the plasticity of the Q&P steel. Recent studies have indicated that austenite reverted transformation (ART) heat treatment comprising prequenching, intercritical annealing, and accelerated cooling can refine the retained austenite in medium Mn steel and improve the stability of the retained austenite [6,7,8]. During intercritical annealing, the prequenching martensite was gradually transformed into a duplex microstructure of superfine lath ferrite and austenite. Inspired by ART treatment, Zhang et al. [9] combined prequenching and Q&P heat treatment and proposed a new heat treatment named QQ&P treatment, a process that can refine the size of austenite before quenching and improve its thermal stability. Compared with the Q&P sample prepared by traditional Q&P treatment, the QQ&P sample has a higher volume fraction and a finer retained austenite grain size, thereby achieving a better combination of strength and ductility. Clearly, delaying austenite coarsening during annealing is the key factor to improving the retained austenite content of the QQ&P sample. The intercritical annealing temperature will directly affect ART, thus changing the volume fraction and size of austenite before quenching. The carbon partitioning and the stability of retained austenite are significantly influenced by the morphology and volume fraction of austenite after intercritical annealing [10,11]. Therefore, the annealing temperature has a significant effect on the volume fraction and morphology of retained austenite in QQ&P steel.

At present, there have been studies on the effect of annealing temperature on the microstructures and mechanical properties of traditional Q&P steel [11,12,13]. According to these studies, the annealing temperature has a great effect on morphology, carbon content, and volume fraction of retained austenite in Q&P steel. However, after austenitizing and quenching processes, the initial microstructure is transformed to martensite lath, obviously different from that of traditional Q&P steel [9]. The initial microstructures affect the formation kinetics and growth of austenite during annealing [14,15]. Therefore, the effect of annealing temperature on the microstructures of QQ&P steel may be significantly different from that on the microstructures of Q&P steel. However, there are few studies on the influence of annealing temperature on the microstructure evolution of QQ&P steel until now, and the influence of the annealing temperature on the mechanical properties of QQ&P steel is not clear. An in−depth study is needed. This is the novelty of this paper.

In this paper, we comparatively study the microstructures’ evolution and mechanical properties of traditional Q&P samples and QQ&P samples at different annealing temperatures to demonstrate the influence of the annealing temperature on microstructures and mechanical properties of QQ&P steel. In addition, the effect of the morphology of structures on strain hardening behavior and total elongation is explored.

## 2. Experimental Procedure

**Figure 1:**
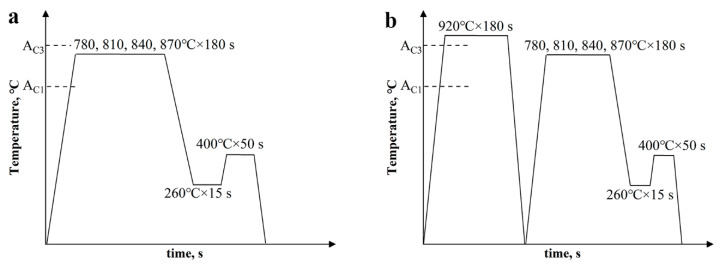
Schematic diagrams of (**a**) Q&P and (**b**) QQ&P heat treatments.

The experimental cold−rolled (CR) sheets were prepared by subjecting the ~2 mm thickness commercial hot−rolled (HR) sheets to 35% cold reduction in thickness. The chemical compositions of the sheet are shown in Table 1. In designing an appropriate heat−treatment process, the expansion curve of CR sheet during heating and cooling was measured with a Gleeble 3500 type dilatometer, and the A_c1_ (starting temperature of austenite formation during heating), A_c3_ (end temperature of austenite formation during heating), M_s_ (starting temperature of martensite formation during cooling) and M_f_ (end temperature of martensite formation during cooling) temperatures of the steel were 706 °C, 909 °C, 379 °C, and 215 °C, respectively. The cold sheets were divided into two groups. The first group (named Q&P) was annealed at 780 °C, 810 °C, 840 °C, and 870 °C for 3 min, respectively, before undergoing an immediate quench (first quenching) to 260 °C for 15 s in a salt bath and another salt bath at 400 °C for 50 s. Lastly, the samples were quenched (second quenching) in water to room temperature (Figure 1a). The second group (named QQ&P) was treated in the same way as the first group but with a prequenched process involving quenching from 900 °C to room temperature (Figure 1b).

The QQ&P and Q&P samples for the scanning electron microscope (SEM, Nova nano 400, FEI Company, Hillsboro, OR, USA) studies were mechanically polished and then etched in 4 vol% initial solution for 10 s. The electron backscatter diffraction (EBSD, Apreo S HiVac, Thermofisher, Waltham, MA, USA) samples were electrochemically etched at 5% perchloric acid alcohol at 25 °C with a current of 0.6 A and a voltage of 28 V for about 30 s. EBSD measurement was carried out at 15 kV and at a step size of 50 nm. The transmission electron microscope (TEM, JEM-2100, Tokyo, Japan) samples were thinned to a thickness of 60 μm and then punched into 3 mm diameter discs. The discs were finally electro-polished with a twin−jet machine at −25 °C in a solution of perchloric acid and alcohol. The volume fractions of retained austenite in samples were measured by X-ray diffraction (XRD, Panslytical, Almelo, The Netherlands) with Cu Kα radiation. The integrated intensities of the (200)γ, (220)γ, (311)γ, (200)α, and (211)α peaks were used to quantify the volume fraction of retained austenite by Equation (1) [16].

(1) $$V=\frac{1}{1+G\left(\frac{I_{\alpha }}{I_{\gamma }}\right)}$$

where *V* is the volume fraction of retained austenite, $I_{\gamma }$ is the integrated intensity of the FCC reflection peaks, and *I_α_* is the integrated intensity of the BCC reflection peaks.

The carbon content of retained austenite is quantified by Equation (2) [17].

(2) $$\alpha_{\gamma }=0.3556+0.00453x_{C}+0.000095x_{Mn}$$

where $\alpha_{\gamma }$ is the lattice constant of austenite, nm; $x_{C}$ and $x_{Mn}$ correspond to the mass fractions of carbon and manganese in austenite, %, respectively.

The tensile samples after heat treatment were machined to have a profile of 93 × 20 mm. The gage length and width of samples were 30 mm and 12.5 mm, respectively. Tensile tests were conducted at a strain rate of 5 × 10^−4^ s^−1^ using a CMT5304 (Shenzhen SUNS Technology Stock Co., Ltd., Shenzhen, China) tensile machine at room temperature. Each sample was tested three times to obtain an average value for each mechanical property.

## 3. Results

### 3.1. Microstructures of QQ&P and Q&P Samples

**Figure 2:**
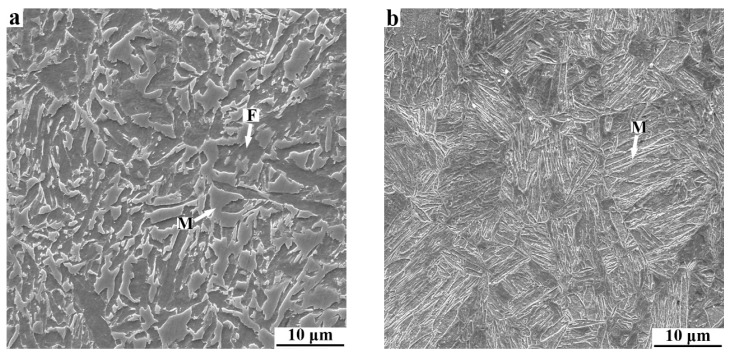
SEM micrographs of (**a**) CR and (**b**) prequenching C-Si-Mn sheets. F and M refer to ferrite and martensite, respectively.

The SEM micrographs reflecting the initial microstructures of CR and prequenching C−Si−Mn sheets before Q&P treatment are shown in Figure 2. The microstructures of the CR sheet are mainly composed of ferrite and martensite (Figure 2a). After the full process of austenitizing and prequenching, the microstructure of the sheet is predominantly martensite laths (Figure 2b).

**Figure 3:**
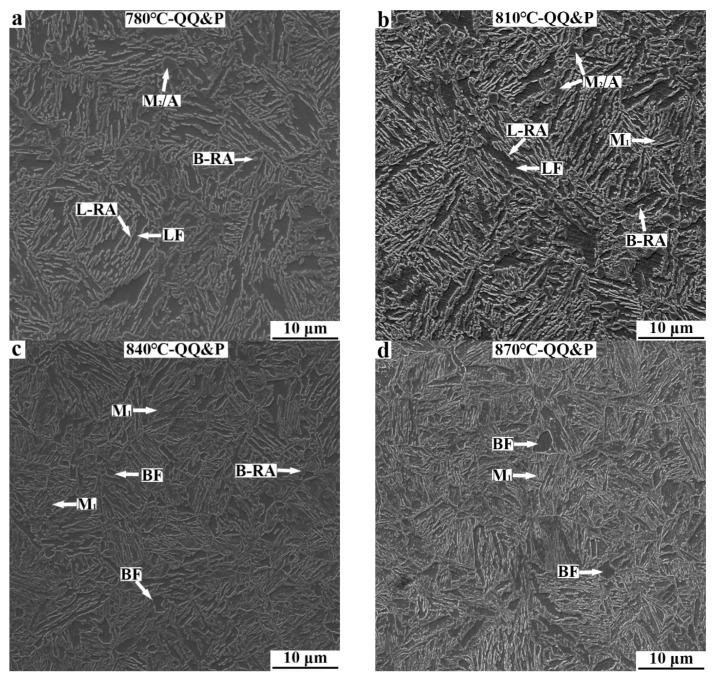
SEM micrographs of QQ&P samples at annealing temperatures of (**a**) 780 °C, (**b**) 810 °C, (**c**) 840 °C, and (**d**) 870 °C. LF and BF refer to ferrite with lath morphology and blocky morphology, respectively. M_1_ and M_2_/A refer to primary martensite and secondary martensite/retained austenite. L-RA and B-RA refer to retained austenite with lath morphology and blocky morphology, respectively.

The SEM micrographs of QQ&P samples are presented in Figure 3. M_1_ in the figures refers to the primary martensite that forms during the first quenching, a process followed by carbon depletion and tempering in the partitioning region. LF and BF refer to lath ferrite and blocky ferrite, respectively. L-RA and B-RA refer to retained austenite with lath morphology and blocky (or granular) morphology, respectively. M_2_/A island refers to secondary martensite/carbon-enriched retained austenite. M_2_ forms during the second quenching to room temperature. The ferrite, M_1_, retained austenite, and M_2_/A island can be identified by their different responses [18,19]. When the prequenched sample is annealed at 780 °C (Figure 3a), the microstructures of samples mainly consist of ferrite, retained austenite, and M_2_/A island. The morphology of ferrite is mainly lath-shaped. The retained austenite distributed along the longitudinal direction of lath ferrite is mainly lath-shaped. Some retained austenite distributed in the interior of lath ferrite is block−shaped. The microstructures of the 810 °C-QQ&P sample are similar to those of the 780 °C-QQ&P sample, except that a small amount of M_1_ is formed (Figure 3b). With the annealing temperature increased to 840 °C, the content of M_1_ increases, and the content of ferrite decreases. Different from the 780 °C-QQ&P sample and the 810 °C-QQ&P sample, the 840 °C-QQ&P sample has more blocky ferrite grains. In addition, the M_2_/A island basically disappears (Figure 3c). When the annealing temperature increases to 870 °C, the content of M_1_ increases further, the content of ferrite decreases, and the ferrite is mainly block−shaped (Figure 3d).

**Figure 4:**
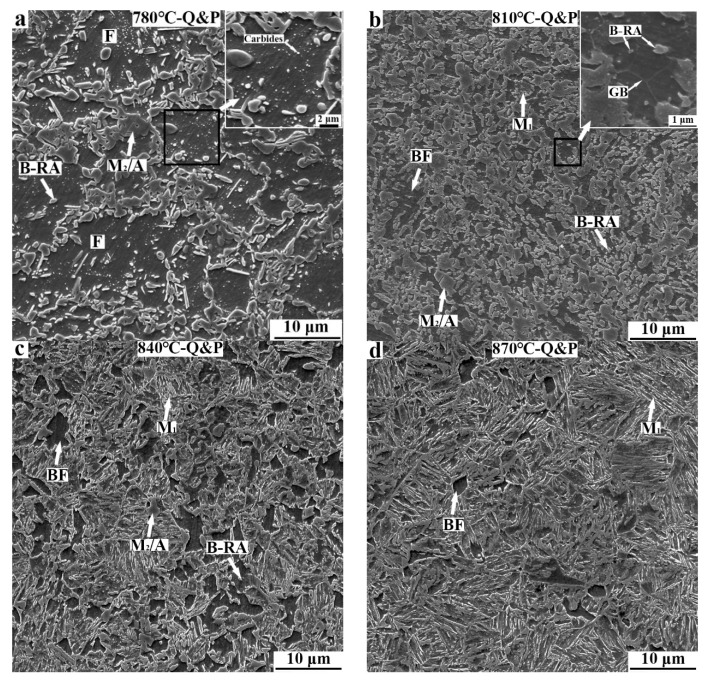
SEM micrographs of Q&P samples at annealing temperatures of (**a**) 780 °C, (**b**) 810 °C, (**c**) 840 °C, and (**d**) 870 °C. GB in (**b**) refers to grain boundary. F and BF refer to ferrite with irregular morphology and blocky morphology, respectively.

SEM micrographs of Q&P samples at different annealing temperatures are shown in Figure 4. When the CR sample is annealed at 780 °C, its microstructures consist of ferrite, carbides, retained austenite, and M_2_/A island. The ferrite in the sample mainly presents an irregular coarse morphology, and the retained austenite mainly presents a blocky morphology. The carbides are distributed inside the ferrite grains. In addition, the number of M_2_/A islands is obviously higher than that in the 780 °C-QQ&P sample (Figure 4a). When the annealing temperature is increased to 810 °C, the ferrite grains mainly present the blocky type, and their size significantly decreases. As the high−magnification micrograph shows, ferrite grains undergo obvious recrystallization. Furthermore, the number of carbides decreases, and a small amount of M_1_ is discovered (Figure 4b). When the CR sample is annealed at 840 °C, the content of M_1_ increases, and some M_2_/A islands remain (Figure 4c). When the annealing temperature increases to 870 °C, the contents of ferrite and the M_2_/A island decrease. The microstructures are basically the same as that of the 870 °C QQ&P sample (Figure 4d).

**Figure 5:**
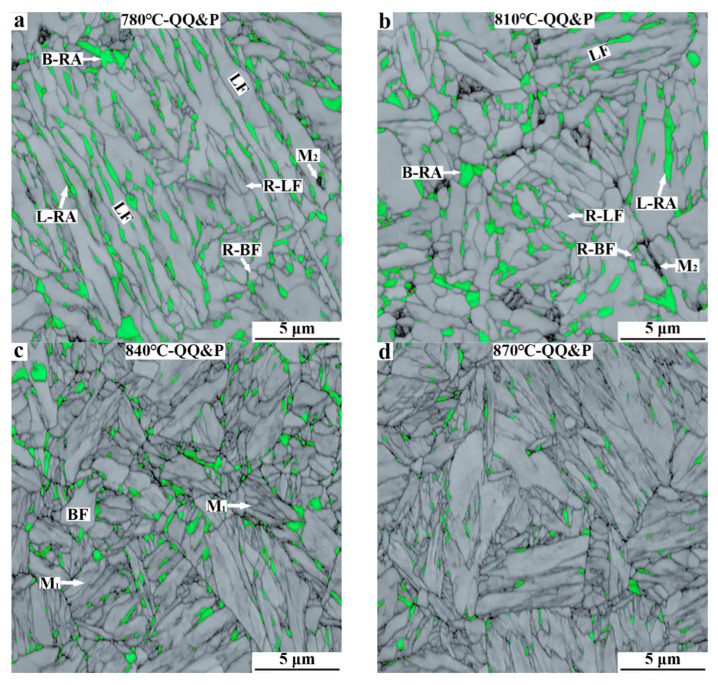
EBSD micrographs of QQ&P samples at annealing temperatures of (**a**) 780 °C, (**b**) 810 °C, (**c**) 840 °C, and (**d**) 870 °C. R−LF and R−BF in (**a**,**b**) refer to recrystallized ferrite with lath morphology and blocky morphology, respectively. M_2_ refers to secondary martensite austenite.

The image quality micrographs of QQ&P samples analyzed by EBSD are shown in Figure 5. Austenite with an FCC crystal structure is highlighted in green. Ferrite and M_1_ with BCC crystal structures are shown in grey. The light grey refers to ferrite, and dark grey refers to M_1_. Considering the high defect densities and large quantities of substructures that contribute to low image quality, the dark region represents M_2_ [20]. R-LF and R-BF in Figure 5a,b refer to recrystallized lath ferrite and recrystallized blocky ferrite, respectively. When the prequenched sample is annealed at 780 °C, the ferrite in the sample is mainly lath−shaped. Meanwhile, a small amount of recrystallized blocky ferrite grains are also discovered. A large number of lath retained austenite and some blocky retained austenite are distributed along the lath ferrite and at grain boundaries of blocky ferrite, respectively. In addition, a small amount of M_2_ is present in the sample (Figure 5a). When the annealing temperature increases to 810 °C, a large number of recrystallized lath and blocky ferrite grains and a small amount of M_2_ are discovered. However, the amount of blocky retained austenite in the 810 °C-QQ&P sample is obviously greater than that in the 780 °C-QQ&P sample (Figure 5b). When the annealing temperature is 840 °C, the contents of ferrite and lath retained austenite significantly decrease. A large number of M_1_ laths are discovered and thin−film retained austenite is distributed between martensite laths. In addition, some blocky retained austenite is distributed at the boundaries of prior austenite grains (Figure 5c). When the annealing temperature is increased to 870 °C, the retained austenite in the sample has a morphology similar to that in the 840 °C-QQ&P sample, but its content decreases (Figure 5d).

**Figure 6:**
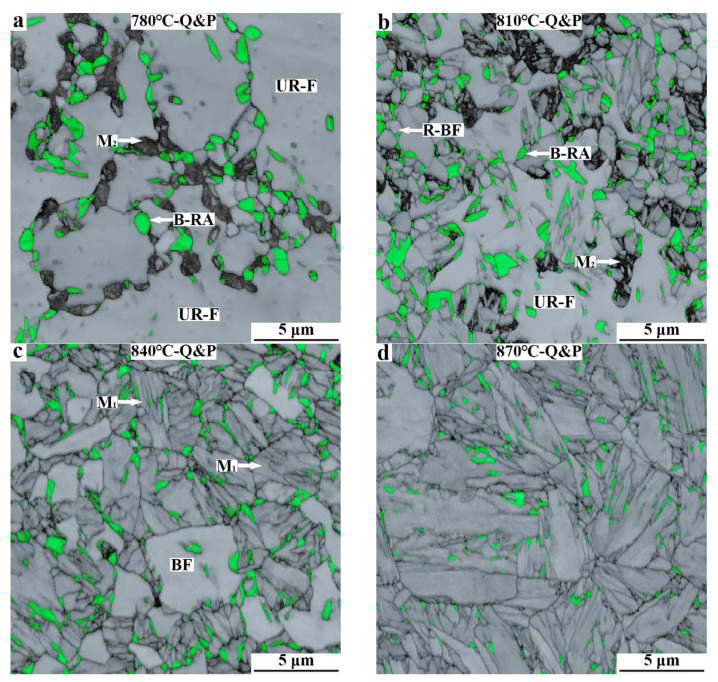
EBSD micrographs of Q&P samples at annealing temperatures of (**a**) 780 °C, (**b**) 810 °C, (**c**) 840 °C, and (**d**) 870 °C. UR−F in (**a**,**b**) refers to unrecrystallized ferrite.

The image quality micrographs of Q&P samples are shown in Figure 6. UR−F in Figure 6a,b refers to unrecrystallized ferrite. The results indicate that the morphology, grain size, and distribution of ferrite and retained austenite in Q&P samples are different from those in QQ&P samples. Such differences can be summarized in the following aspects (Figure 6a). Firstly, when the CR sheet is annealed at 780 °C, the recovery and growth of ferrite occur during annealing, which results in the coarse and irregular shape of ferrite in the 780 °C−Q&P sample. With the annealing temperature increased to 810 °C, the ferrite is partially recrystallized, and the recrystallized ferrite is mainly blocky (Figure 6b). Secondly, the retained austenite in the 780 °C-Q&P and 810 °C-Q&P samples is mainly blocky and distributed in the interior or at the boundaries of ferrite grains (Figure 6a,b). Lastly, the contents of M_2_ in the 780 °C−Q&P sample and the 810 °C-Q&P sample are obviously higher than that in the QQ&P samples. When the annealing temperature is increased to 870 °C, the morphologies of ferrite and retained austenite are similar to that in the 870 °C-QQ&P sample (Figure 6c,d).

**Figure 7:**
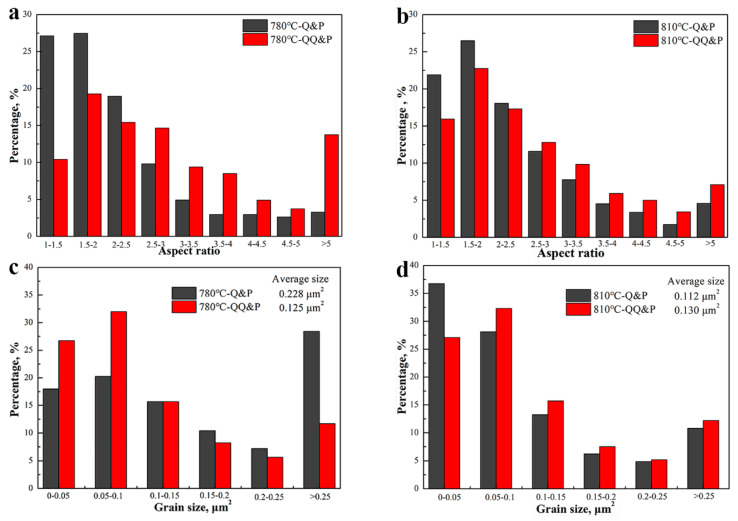
Statistical distributions of (**a**,**b**) aspect ratio and (**c**,**d**) size of retained austenite grains that are simulated into ellipses in Q&P and QQ&P samples at annealing temperatures of (**a**,**c**) 780 °C and (**b**,**d**) 810 °C.

To further analyze the amount of lath and blocky retained austenite in QQ&P and Q&P samples, the retained austenite grains in QQ&P and Q&P samples at annealing temperatures of 780 °C and 810 °C are simulated into ellipses using software, and the distributions of aspect ratio and grain size are counted, as shown in Figure 7. Compared with the blocky retained austenite, the lath retained austenite has a larger aspect ratio. The results indicate that the percentages of retained austenite with an aspect ratio over 2.5 in the 780 °C-QQ&P sample are higher than those in the 780 °C−Q&P sample (Figure 7a). Similar conditions are indicated when the annealing temperature increases to 810 °C (Figure 7b). These results indicate that the 780 °C-QQ&P and 810 °C-QQ&P samples have more lath retained austenite than the Q&P samples. In addition, the percentage of retained austenite with an aspect ratio below 2.5 in the 810 °C-QQ&P sample is obviously higher than that in the 780 °C-QQ&P sample, which means that as the annealing temperature increases from 780 °C to 810 °C, the percentage of blocky retained austenite increases, and the percentage of lath retained austenite decreases. The size distributions of retained austenite in QQ&P and Q&P samples at annealing temperatures of 780 °C and 810 °C are shown in Figure 7c and d, respectively. The results indicate that when annealed at 780 °C, the Q&P sample has an obviously higher percentage of retained austenite, with a grain size over 0.25 μm^2^, compared to the QQ&P sample (Figure 7c). Meanwhile, the average grain size of retained austenite in the QQ&P sample is significantly finer than that in the Q&P sample (0.228 μm^2^ vs. 0.125 μm^2^). When the annealing temperature increases to 810 °C, the average grain size of retained austenite in the Q&P sample decreases significantly (Figure 7d). However, compared with the grain size of retained austenite in the 780 °C-QQ&P sample, the grain size of retained austenite in the 810 °C-QQ&P sample increases slightly (0.125 μm^2^ vs. 0.130 μm^2^).

**Figure 8:**
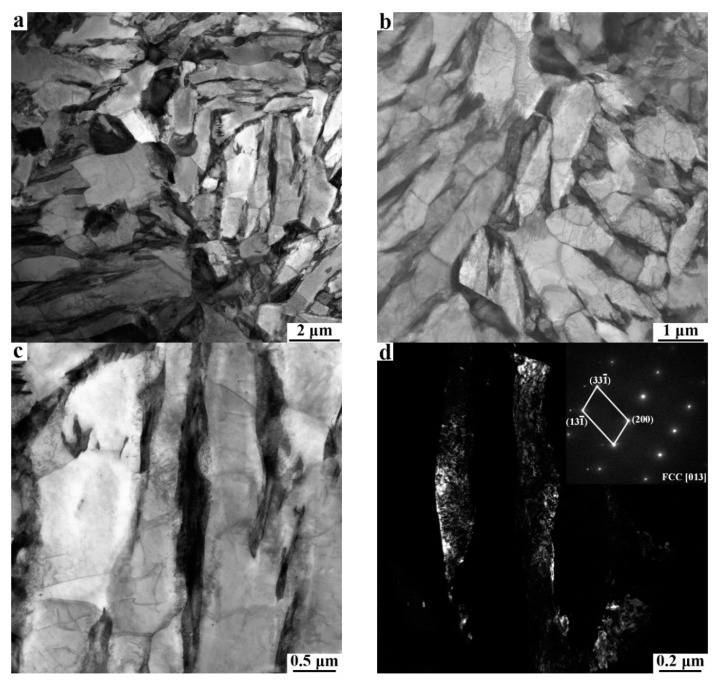
(**a**,**b**) Typical TEM micrographs of the 810 °C-QQ&P sample. Micrographs (**c**,**d**) are the bright field and the dark field of L−RA, respectively.

**Figure 9:**
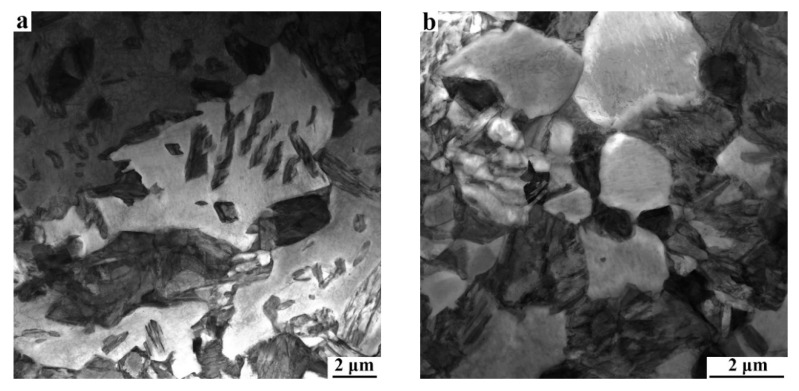

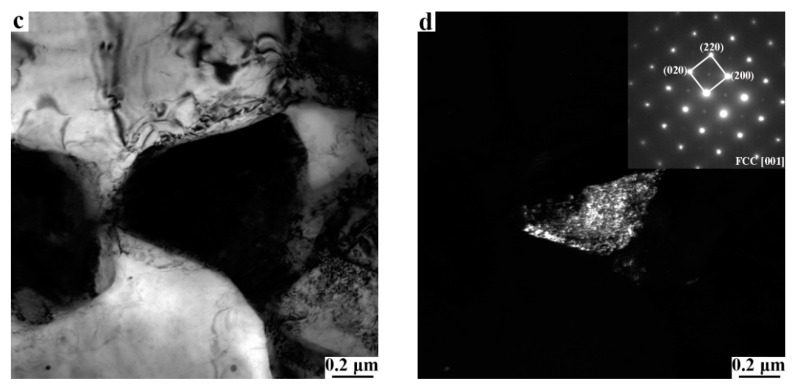
(**a**,**b**) Typical TEM micrographs of the 810 °C-Q&P sample. Micrographs (**c**,**d**) are the bright field and dark field of B−RA, respectively.

Figure 8 and Figure 9 show the typical TEM microstructures of the 810 °C-QQ&P sample and the 810 °C-Q&P sample, respectively. The TEM micrographs indicate that the ferrite is mainly lath-shaped (Figure 8a). Most lath ferrite recrystallizes and forms finer ferrite grains. The recrystallized ferrite grains are lath and blocky (Figure 8b), which is consistent with the EBSD results (Figure 5b). The retained austenite along the longitudinal direction of the lath ferrite is lath−shaped (Figure 8c,d). Unlike the case in the 810 °C-QQ&P sample, coarse, irregular, unrecrystallized ferrite grains and fine, blocky, recrystallized ferrite grains are simultaneously found in the 810 °C-Q&P sample (Figure 9a,b). Retained austenite distributed at the grain boundary of blocky ferrite grains is mainly blocky (Figure 9c,d).

**Figure 10:**
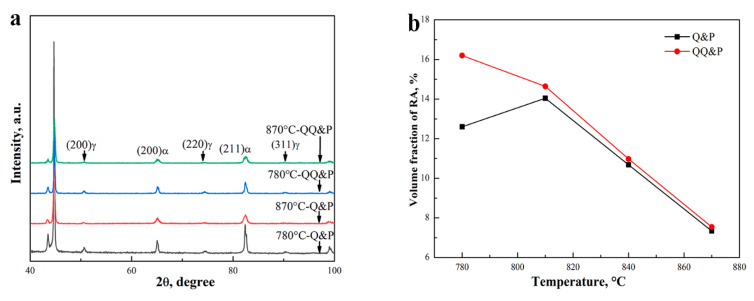

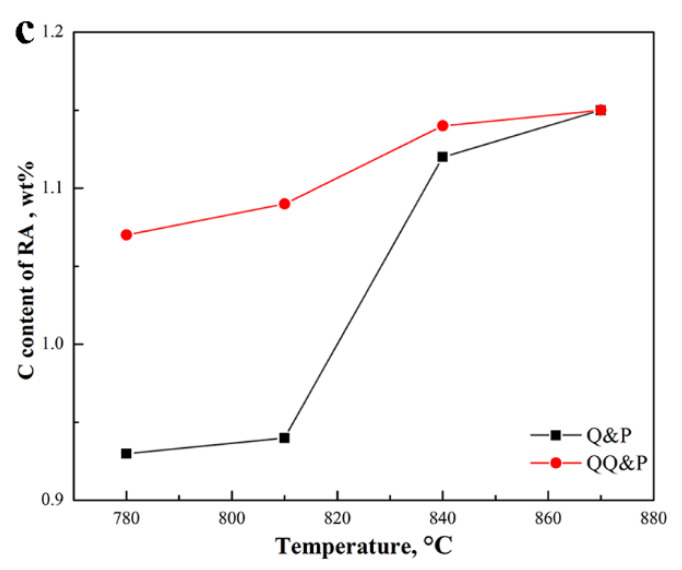
(**a**) XRD patterns of Q&P and QQ&P samples at annealing temperatures of 780 °C and 810 °C, (**b**) the volume fraction, and (**c**) carbon content of retained austenite in Q&P and QQ&P samples as a function of annealing temperature.

The comprehensive XRD patterns of QQ&P and Q&P samples at annealing temperatures of 780 °C and 870 °C are shown in Figure 10. The patterns indicate that the intensities of (200)γ, (220)γ, and (311)γ in 780 °C Q&P and 780 °C QQ&P samples are higher than their counterparts in the 870 °C-Q&P sample and the 870 °C-QQ&P sample. In addition, the intensities of (200)α and (211)α in the 780 °C-Q&P sample are obviously higher than that in the 780 °C-QQ&P sample. The contents of retained austenite in QQ&P and Q&P samples at different annealing temperatures are summarized in Figure 10b. The results show that the volume fraction of retained austenite in QQ&P samples decreases with the increase in annealing temperature. However, retained austenite in Q&P samples shows a different trend. The volume fraction of retained austenite in Q&P samples first increases when the annealing temperature is increased from 780 °C to 810 °C but then decreases when the temperature is further increased. The volume fraction of retained austenite in QQ&P samples is higher than that in Q&P samples when the annealing temperature is either 780 °C or 810 °C. However, when the annealing temperature increases to 840 °C and 870 °C, the volume fraction of retained austenite in QQ&P samples is similar to that in Q&P samples. The carbon contents of retained austenite in QQ&P and Q&P samples at different annealing temperatures are summarized in Figure 10c. As the results indicate, the carbon content of retained austenite in both the QQ&P and Q&P samples increases with the annealing temperature. When annealed at 780 °C and 810 °C, QQ&P samples have obviously more carbon in retained austenite than the Q&P samples.

### 3.2. Mechanical Properties of QQ&P and Q&P Samples

**Figure 11:**
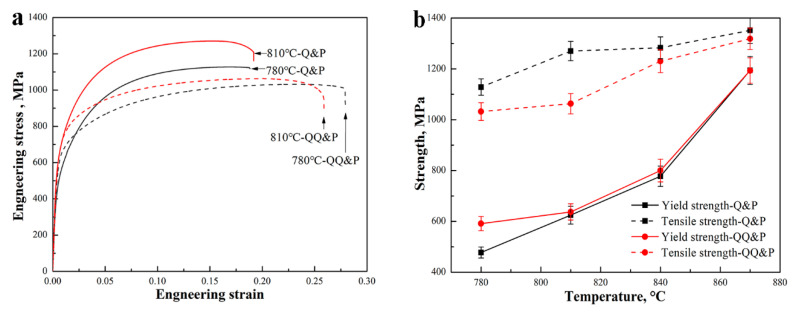

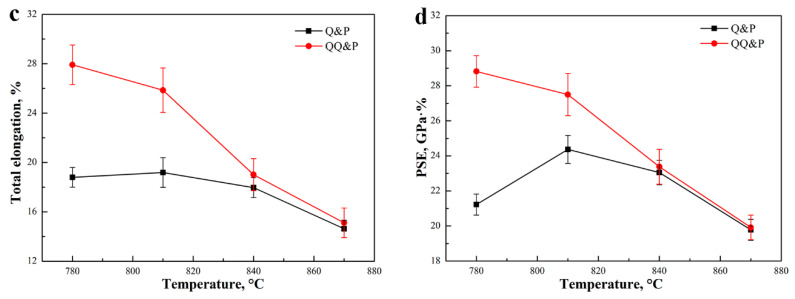
(**a**) The engineering stress–strain plots of QQ&P and Q&P samples at annealing temperatures of 780 °C and 810 °C, (**b**) strength, (**c**) total elongation, and (**d**) product of strength and elongation (PSE) of QQ&P and Q&P samples as a function of annealing temperature.

The typical engineering stress–strain plots of QQ&P and Q&P samples at annealing temperatures of 780 °C and 810 °C are shown in Figure 11a. The mechanical properties of QQ&P and Q&P samples at different temperatures are summarized in Figure 11b–d. The yield strength and tensile strength of QQ&P and Q&P samples both increase with the temperature. Compared with Q&P samples, the QQ&P samples exhibit higher yield strength when annealed at 780 °C. When the annealing temperature is increased, the yield strengths of QQ&P samples and Q&P samples are similar. The tensile strengths of QQ&P samples at annealing temperatures of 780 °C and 810 °C are lower than those of Q&P samples. With the further increase in annealing temperature, the difference in tensile strength between QQ&P samples and Q&P samples decreases (Figure 11b). Both the total elongation and the product of strength and elongation (PSE) of QQ&P samples decrease with the increasing annealing temperature. Nevertheless, the total elongation and the PSE of Q&P samples first increase and then decrease with the increasing annealing temperature. In addition, the total elongations and PSE of QQ&P samples at annealing temperatures of 780 °C and 810 °C are obviously higher than those of the Q&P samples. The maximum PSE of the QQ&P sample and Q&P sample is 28.8 GPa·% and 24.4 GPa·%, respectively. With a further increase in annealing temperature, the total elongation and PSE of QQ&P samples and Q&P samples are similar (Figure 11c,d).

## 4. Discussion

### 4.1. The Effect of Annealing Temperature on the Morphology of Ferrite in QQ&P and Q&P Samples

After the prequenching process, the initial structure is mainly martensite laths. During intercritical annealing, austenite reversed transformation (ART) that consumes the carbides and dislocations of martensite will occur, and the ferrite lath will form [21]. The study by Wang et al. [22] indicated that all the orientations of lath ferrite after ART annealing in medium Mn steel are inherited from those of the martensite lath. Therefore, it can be concluded that the morphologies of lath ferrite in 780 °C-QQ&P and 810 °C-QQ&P samples were inherited from the prequenching martensite laths. Unlike the case in the 780 °C-QQ&P sample, in the 810 °C-QQ&P sample, the higher annealing temperature increases the diffusion rate of atoms and promotes the migration of grain boundaries [23], which results in more pronounced recrystallization and growth of ferrite. Because of the effect of the morphology of lath ferrite, part of the recrystallized ferrite grains remains lath−shaped and does not grow into a blocky shape. With an increase in the annealing temperature, more martensite laths will be transformed into austenite during annealing. In addition, due to the higher annealing temperature, ferrite is more prone to recrystallization and growth, leading to the transformation of lath ferrite to blocky ferrite.

When annealed at 780 °C and 810 °C, Q&P samples and QQ&P samples have quite different morphologies of ferrite. This could be attributed to their different initial microstructures before annealing. The initial microstructures of the CR sheet are mainly deformed ferrite and martensite. After cold-rolling, dislocations and deformation bands will form in ferrite grains [24], increasing the stored energy. The stored energy can provide a driving force for the ferrite recovery and recrystallization upon intercritical annealing. When the annealing temperature is low, it is difficult for ferrite to recrystallize, and the ferrite grains are mainly recovered and grow, resulting in their coarse and irregular morphology in the 780 °C-Q&P sample. When the annealing temperature is increased to 810 °C, ferrite recrystallizes readily during annealing, which significantly refines the ferrite grains. Due to the high storage energy of the CR sheet, recrystallized ferrite can grow directly into a blocky shape. This is the reason why the morphology of recrystallized ferrite in the 810 °C-Q&P sample is blocky.

### 4.2. The Effect of Annealing Temperature on the Morphology, Size, and Content of Retained Austenite in QQ&P and Q&P Samples

As mentioned above, the prequenching sample with an initial structure of martensite laths will undergo ART intercritical annealing, which can be summarized as follows: (1) carbide precipitation and austenite nucleation at the beginning; (2) the formation of austenite laths between the martensite laths, during which carbides and dislocations of martensite are consumed; and (3) complete formation of the duplex structure of ultrafine lath austenite and ferrite [21]. Compared with blocky austenite, lath austenite has a higher ratio of surface to volume. This can reduce the carbon diffusion path, thereby improving the homogenization degree of carbon in austenite grains. With a high carbon content, the lath austenite in 780 °C-QQ&P and 810 °C-QQ&P samples exhibits better thermal stability and can be retained at room temperature. Due to the growth of some recrystallized ferrite into the blocky shape in the 810 °C-QQ&P sample, the percentage of lath retained austenite between ferrite laths decreases (Figure 7a,b). With the annealing temperature increased to 870 °C, most prequenched martensite laths are transformed into austenite, leading to a significant decrease in ferrite content. Most austenite with low thermal stability is transformed to M_1_ during the first quenching. A small amount of the untransformed austenite with high thermal stability is mainly distributed in carbon−rich areas, such as boundaries of prior austenite grains or interfaces of martensite laths. After partitioning, the blocky austenite distributed at the boundaries of the prior austenite grains and the thin film austenite distributed between the martensite laths can be retained at room temperature.

Austenite distributed at the boundaries of blocky ferrite grains tends to grow into a blocky shape [25], explaining why the retained austenite is mainly blocky in 780 °C-Q&P and 810 °C-Q&P samples. It should be noted that the grain size of retained austenite in the 810 °C-Q&P sample is significantly finer than that in the 780 °C-Q&P sample. This is related to finer recrystallized ferrite grains in the 810 °C-Q&P sample. Small-sized recrystallized ferrite grains can inhibit the growth of adjacent austenite grains and refine the austenite grains [26]. With the annealing temperature increased to 870 °C, the CR structures are mostly transformed into austenite, and the effect of the initial structures between the QQ&P sample and the Q&P sample on the morphology of retained austenite can be ignored.

The volume fraction of retained austenite in the Q&P steel is closely related to the thermal stability of austenite, which is determined by the carbon enrichment in austenite [1]. The effects of annealing temperature on the volume fraction of retained austenite in QQ&P and Q&P samples are closely related to the thermal stability of austenite after annealing. When the annealing temperature is low, less austenite and more ferrite form during annealing, resulting in higher carbon content in austenite after annealing. Most austenite with high thermal stability can be retained at room temperature without martensitic transformation in the quenching stages. With the increase in the annealing temperature, the content of austenite increases, and the content of ferrite decreases during annealing, leading to the decrease in carbon content in austenite after annealing. Abundant austenite with low thermal stability is transformed to M_1_ during the first quenching, resulting in the decrease in volume fraction of retained austenite in QQ&P samples with the increasing annealing temperature. After carbon partitioning from M_1_ laths, the carbon content in the untransformed austenite increases, and the austenite can be retained at room temperature. This is the reason why the carbon content increases with the annealing temperature. However, the volume fraction of retained austenite in Q&P samples shows the opposite trend when the annealing temperature is increased from 780 °C to 810 °C. This can be attributed to the large number of carbides formed in the 780 °C-Q&P sample. The formation of carbides will consume the carbon in steel and reduce the carbon enrichment in austenite, thus reducing the thermal stability of austenite [27,28]. The retained austenite with low thermal stability can be easily transformed into secondary martensite during quenching to room temperature, resulting in a decreased retained austenite content in the 780 °C-Q&P sample.

Furthermore, the volume fraction and carbon content of retained austenite in QQ&P samples are both higher than those in Q&P samples when annealed at 780 °C and 810 °C. There are three reasons for this. First, QQ&P samples have undergone austenitizing and prequenching treatment before intercritical annealing, which indicates the even distribution of carbon in the prequenching structure before annealing. Due to the preferential precipitation of carbides in the carbon−rich zone, the uniform distribution of carbon in the prequenching structure can inhibit the precipitation of carbides during intercritical annealing, thereby improving the carbon content and volume fraction of retained austenite in QQ&P samples. Second, during the ART annealing stage, a large amount of austenite in the prequenched samples will nucleate and grow into a lath shape between the martensite laths [22]. Compared with blocky austenite, lath austenite has a larger contact area with the adjacent martensite laths, which is conducive to the diffusion of carbon from martensite to austenite, thus improving the carbon content and thermal stability of austenite [9]. Last, compared with blocky austenite, lath austenite has a higher ratio of surface to volume. This can reduce the carbon diffusion path, thereby improving the homogenization degree of carbon in austenite grains. The lath austenite with a more uniform carbon distribution can inhibit its transformation into M_2_ during the second quenching process so that more austenite can be retained at room temperature. This explains the higher content of retained austenite and the lower content of M_2_ in the 780 °C-QQ&P and 810 °C-QQ&P samples. With the annealing temperature increased to 870 °C, the prequenched structures and cold−rolled structures are basically transformed into austenite after annealing. The effect of prequenching on the volume fraction of retained austenite can be ignored.

### 4.3. The Relationship between Microstructures and Mechanical Properties of QQ&P Samples and Q&P Samples

Martensite, as a hard phase, can increase the strength of steel but decrease its plasticity. Ferrite, as a soft phase, undergoes plastic deformation that can improve the plasticity but reduce the strength of the steel. Furthermore, the TRIP effect of retained austenite is the key factor in improving the plasticity of advanced high−strength steel (AHSS) [29,30,31]. Both the yield strength and the tensile strength of QQ&P and Q&P samples increase with the annealing temperature. This is related to the decrease in ferrite content and the increase in primary martensite content. The trend of total elongation of QQ&P samples and Q&P samples with the increasing annealing temperature is consistent with the trend of retained austenite content with the increasing annealing temperature. This confirms that the retained austenite content has an important effect on the elongation of AHSS. In addition, when annealed at 780 °C and 810 °C, QQ&P samples have obviously better total elongations than Q&P samples. To explore why the QQ&P samples have better total elongations, we discuss the relationship between microstructures and elongation of the 810 °C-QQ&P sample and the 810 °C-Q&P sample with consideration of the following aspects.

#### 4.3.1. The Influence of Microstructures on Uniform Elongations of QQ&P and Q&P Samples

**Figure 12:**
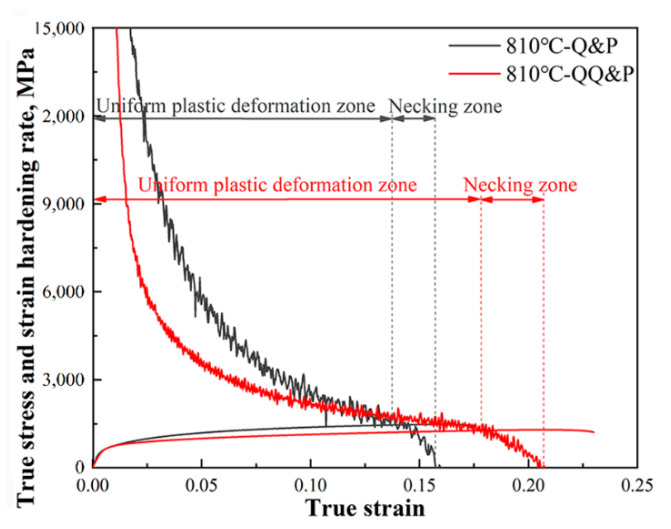
True stress–strain and strain hardening rate plots of QQ&P and Q&P samples at the annealing temperature of 810 °C.

The trend of the strain hardening rate is closely related to the microstructure evolution during deformation. To illustrate the plasticity mechanism of 810 °C-QQ&P and 810 °C-Q&P samples, we display the true stress–strain plots and strain hardening rate (SHR) plots of 810 °C-QQ&P and 810 °C-Q&P samples in Figure 12. When the SHRs are equal to the true stress, necking occurs. The criterion for necking is shown as Equation (3) [32]:

(3) $${d\sigma }_{T}/{d\varepsilon }_{T}=\sigma_{T}\;\mathrm{at}\;\varepsilon_{T}=\varepsilon_{U}$$

where ${d\sigma }_{T}/{d\varepsilon }_{T}$ is SHR, $\sigma_{T}$ is the true stress and $\varepsilon_{T}$ is the true strain. $\varepsilon_{U}$ is the true strain value that corresponds to the beginning of necking. The SHR plots are divided into two stages: the uniform plastic deformation stage and the necking stage. The uniform plastic deformation stage is mainly linked with the ferrite plastic deformation and the TRIP effect of retained austenite [11]. The results indicated that the range of uniform plastic deformation stage for the 810 °C-QQ&P sample is significantly larger than that for the 810 °C−Q&P sample (Table 2) for the following reasons. First, compared with the 810 °C-Q&P sample, the 810 °C-QQ&P sample has a higher content of retained austenite, which is beneficial to enhancing the TRIP effect [31] and improving the uniform elongation of the 810 °C-QQ&P sample. Second, the 810 °C-QQ&P sample has two different forms of retained austenite: blocky and lath. The mechanical stability of the lath retained austenite is generally better than that of the blocky retained austenite [10]. Previous studies have shown that the retained austenite with various mechanical stability in Q&P steel is conducive to the continuous transformation of retained austenite [33,34,35], thereby improving the uniform elongation of steel. Third, a study by Sun et al. [36] indicated that there is an angle between some lath ferrite/retained austenite and tensile direction. The lath structures are more prone to instability and rotation during deformation. The rotation of lath structures can release energy and reduce the stress concentration, thereby improving uniform elongation. Last, the 810 °C−Q&P sample has a higher content of M_2_. Since it has not been tempered, the M_2_ has higher strength but lower plastic deformation ability [37]. Therefore, stress concentration can readily occur at its interface, reducing the uniform elongation of the 810 °C−Q&P sample. In conclusion, the better uniform elongation exhibited by the 810 °C-QQ&P sample is related to its higher content of retained austenite, the mechanical stability of the retained austenite, lath structures, and less content of M_2_.

#### 4.3.2. The Effect of Microstructures on Post−Necking Elongation of QQ&P and Q&P Samples

**Figure 13:**
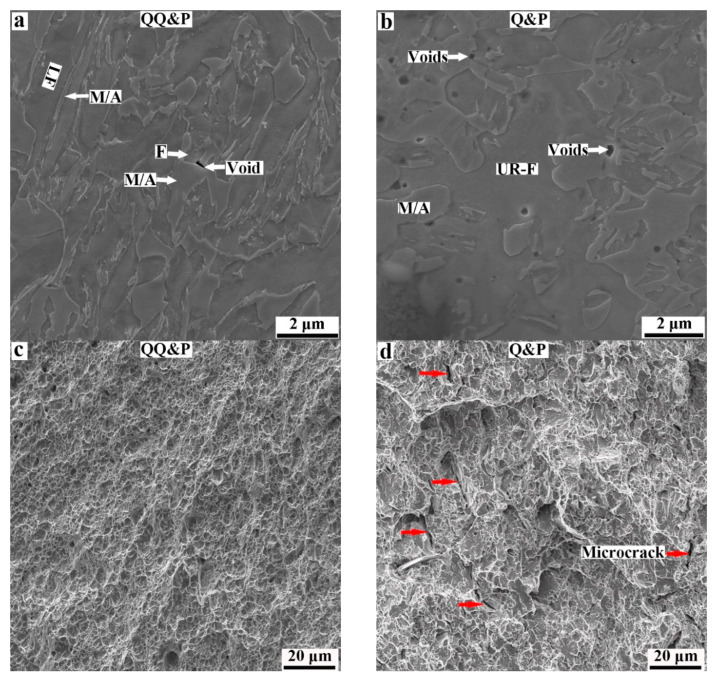
The SEM micrographs of (**a**,**b**) near−fracture zone and (**c**,**d**) fracture surfaces of (**a**,**c**) QQ&P and (**b**,**d**) Q&P samples at the annealing temperature of 810 °C.

The necking stage range for the 810 °C-QQ&P sample is also larger than that for the 810 °C Q&P sample, which means that the 810 °C-QQ&P sample has a better post-necking elongation. The necking stage can be divided into microcrack initiation and crack propagation [38]. During tensile deformation, microcracks are preferentially formed in the stress and strain concentration region [39]. The SEM micrographs of the near-fracture zone and fracture surfaces of 810 °C-QQ&P and 810 °C-Q&P samples are selected to analyze the fracture behavior, as shown in Figure 13. The results indicate that the number of voids and microcracks in the 810 °C−Q&P sample (Figure 13b) is greater than that in the 810 °C-QQ&P sample (Figure 13a). In addition, the fracture surfaces of the two samples indicate that more second cracks are discovered in the 810 °C-Q&P sample (the red arrows in Figure 13c), but tiny and uniform dimples with fewer second cracks are present in the 810 °C-QQ&P sample (Figure 13d). The voids tend to form at the tip of the interface between blocky ferrite and martensite (containing M_2_ and strain-induced martensite), an area prone to stress concentration during deformation. Then, the voids grow along the interface, and the microcracks form. Nevertheless, the lath ferrite and lath retained austenite can smoothen the interface between ferrite and strain−induced martensite, thus slowing down the local stress concentration and delaying the formation of voids and microcracks. This is the reason why the post−necking elongation of the 810 °C-QQ&P sample is better than that of the 810 °C−Q&P sample.

## 5. Conclusions

(1) At annealing temperatures of 780 °C and 810 °C, the ferrite and the retained austenite in QQ&P samples are laths and blocky, while those in Q&P samples are mainly blocky. With the increase in the annealing temperature, the morphology, volume fraction, and carbon content of retained austenite in QQ&P samples become increasingly close to that in Q&P samples.
(2) At annealing temperatures of 780 °C and 810 °C, the volume fraction and carbon content of retained austenite in QQ&P samples are higher than that in Q&P samples. Meanwhile, the total elongation and PSE of QQ&P samples are significantly higher than those of Q&P samples. With the increase in the annealing temperature, the difference between QQ&P samples and Q&P samples in terms of volume fraction and carbon content of retained austenite, total elongation, and PSE decreases gradually.
(3) The higher total elongation and PSE of the QQ&P samples at annealing temperatures of 780 °C and 810 °C are mainly attributed to the fact that lath structures are conducive to carbon diffusion, which can improve the thermal stability and volume fraction of retained austenite in QQ&P samples. Meanwhile, the lath structures can delay the local stress concentration and inhibit the formation of voids and microcracks.

## Figures and Tables

**Table 1:** Chemical compositions of the C−Si−Mn HR sheet (wt.%).

C	Si	Mn	P	S	Ti	N	Al	Fe
0.21	1.69	1.94	0.008	0.0013	0.02	0.0039	0.04	Balanced

**Table 2:** The true strain ranges of uniform plastic deformation stage and necking stage of QQ&P and Q&P samples at annealing temperature of 810 °C.

Sample	Uniform Plastic Deformation Stage	Necking Stage
810 °C-QQ&P	*ε* < 0.178	0.178 < *ε* < 0.207
810 °C-Q&P	*ε* < 0.137	0.137 < *ε* < 0.156

## Data Availability

All data are available from the corresponding author upon reasonable request.
